# Alexithymia in autism: cross-sectional and longitudinal associations with social-communication difficulties, anxiety and depression symptoms

**DOI:** 10.1017/S0033291720003244

**Published:** 2022-06

**Authors:** Bethany F. M. Oakley, Emily J. H. Jones, Daisy Crawley, Tony Charman, Jan Buitelaar, Julian Tillmann, Declan G. Murphy, Eva Loth, The EU-AIMS LEAP Group

**Affiliations:** 1 Department of Forensic and Neurodevelopmental Sciences, Institute of Psychiatry, Psychology & Neuroscience, King's College London, De Crespigny Park, London SE5 8AF, UK; 2 Sackler Institute for Translational Neurodevelopment, Institute of Psychiatry, Psychology & Neuroscience, King's College London, De Crespigny Park, London SE5 8AF, UK; 3 Centre for Brain & Cognitive Development, Birkbeck, University of London, London WC1E 7HX, UK; 4 Department of Psychology, Institute of Psychiatry, Psychology & Neuroscience, King's College London, London, UK; 5 South London and Maudsley NHS Foundation Trust (SLaM), London, UK; 6 Department of Cognitive Neuroscience, Radboud University Nijmegen Medical Center, Donders Institute for Brain, Cognition and Behaviour, Kapittelweg 29, 6525 EN Nijmegen, The Netherlands; 7 Karakter Child and Adolescent Psychiatry University Center, Reiner Postlaan 12, Nijmegen, The Netherlands; 8 Department of Applied Psychology: Health, Development, Enhancement, and Intervention, University of Vienna, Vienna, Austria

**Keywords:** Alexithymia, anxiety, autism, depression, mental health

## Abstract

**Background:**

Alexithymia (difficulties in identifying and describing emotion) is a transdiagnostic trait implicated in social–emotional and mental health problems in the general population. Many autistic individuals experience significant social-communication difficulties and elevated anxiety/depression and alexithymia. Nevertheless, the role of alexithymia in explaining individual variability in the quality/severity of social-communication difficulties and/or anxiety and depression symptoms in autism remains poorly understood.

**Methods:**

In total, 337 adolescents and adults (autism *N* = 179) were assessed for alexithymia on the Toronto Alexithymia Scale and for social-communication difficulties, anxiety and depression symptoms. A total of 135 individuals (autism *N* = 76) were followed up 12–24 months later. We used regression models to establish cross-sectional and longitudinal associations between alexithymia, social-communication difficulties, anxiety and depression symptoms.

**Results:**

Autistic individuals reported significantly higher alexithymia than comparison individuals (*p* < 0.001, *r* effect size = 0.48), with 47.3% of autistic females and 21.0% of autistic males meeting cut-off for clinically relevant alexithymia (score ⩾61). Difficulties in *describing* feelings were particularly associated with current self-reported social-communication difficulties [*p* < 0.001, *β* = 0.57, 95% confidence interval (CI) 0.44–0.67] and predicted later social-communication difficulties (*p* = 0.02, *β* = 0.43, 95% CI 0.07–0.82). Difficulties in *identifying* feelings were particularly associated with current anxiety symptom severity (*p* < 0.001, *β* = 0.54, 95% CI 0.41–0.77) and predicted later anxiety (*p* = 0.01; *β* = 0.31, 95% CI 0.08–0.62).

**Conclusions:**

Our findings suggest that difficulties in identifying *v*. describing emotion are associated with differential clinical outcomes in autism. Psychological therapies targeting emotional awareness may improve social-communication and anxiety symptoms in autism, potentially conferring long-term benefits.

## Introduction

### Background

Alexithymia, literally translated as ‘no words for emotions’, is a subclinical trait with an estimated prevalence of 10% in the general population (Berthoz & Wessa, 2011; Nemiah, Freyberger, & Sifneos, 1976). The construct of alexithymia can be broken down into several facets, including: difficulties in identifying feelings (internally interpreting and differentiating emotions); difficulties in describing feelings (expressing emotions) and externally oriented thinking (focusing attention away from emotions; Bagby, Parker, and Taylor, 1994).

Of note, individuals with high-alexithymic traits are reported to experience significantly more interpersonal difficulties and mental health problems than those with lower levels of alexithymia (Grabe, Spitzer, & Freyberger, 2004; Spitzer, Siebel-Jürges, Barnow, Grabe, & Freyberger, 2005; Vanheule, Desmet, Meganck, & Bogaerts, 2007). Furthermore, alexithymia has been shown to be a significant barrier to intervention success across psychiatry and healthcare (Grabe et al., 2008; Ogrodniczuk, Piper, & Joyce, 2011). In light of these issues, poor self-knowledge of one's internal emotions (i.e. alexithymia) is now recognised as a transdiagnostic mechanism that modulates mental health outcomes in the general population (Fernandez, Jazaieri, & Gross, 2016; National Institute of Mental Health, 2019).

### Alexithymia and social-communication difficulties in autism

Given its intrinsic role in social–emotional processing, it is unsurprising that elevated rates of alexithymia have been reported across a range of neurodevelopmental and neuropsychiatric conditions that affect social and/or emotion understanding (Berthoz, Pouga, & Wessa, 2011; Bird and Cook, 2013). For example, current estimates suggest that at least 40–65% of autistic people experience severe alexithymia (Bird & Cook, 2013; Hobson et al., 2020; Kinnaird, Stewart, & Tchanturia, 2019). Autism is one of the most common, lifelong neurodevelopmental conditions, with a prevalence of ~1.9% (Maenner et al., 2020) – characterised by core traits of social-communication difficulties and restricted and repetitive behaviours (American Psychiatric Association, 2013). In addition, some autistic individuals experience difficulties with emotion awareness and processing. For instance, when recounting personal memories, individuals may tend to focus on objective events, rather than their feelings about those events (Hurlburt, Happé, & Frith, 1994). Similarly, when reflecting on internal emotions (e.g. when asked ‘how do you know when you are happy?’), some autistic individuals consider the observable features of emotions (‘because I am laughing’, ‘because it's my birthday’), over their subjective ‘feeling’ (Rieffe, Meerum Terwogt, & Kotronopoulou, 2007).

Since social-communication difficulties are a core feature of autism, most published studies have focused on associations between alexithymia and difficulties in perceiving and understanding the emotions of others. For example, autistic individuals with higher alexithymic traits experience more difficulties in recognising verbal and non-verbal emotional expressions, compared to those with lower levels of alexithymia (Cook, Brewer, Shah, & Bird, 2013; Heaton et al., 2012; Oakley, Brewer, Bird, & Catmur, 2016) – and these difficulties are associated with everyday social functioning (Trevisan & Birmingham, 2016).

Furthermore, alexithymia in autism has been shown to relate to reductions and/or differences in the production of emotion expressions (Trevisan, Bowering, & Birmingham, 2016; Wagner & Lee, 2008) and emotional empathy towards others (Bird et al., 2010). These skills are critical for accurately interpreting and appropriately responding to social cues and facilitating successful interpersonal relationships (Ekman, 1999) and may, therefore, contribute to increased difficulties in social interactions. Thus, findings to date imply that alexithymia may act as a modifier, exacerbating social-communication difficulties in autism (Mundy, Henderson, Inge, & Coman, 2007).

### Alexithymia and anxiety/depression symptoms in autism

However, far fewer studies have considered the role of alexithymia – particularly difficulties in perceiving one's *own* emotions – in explaining individual variability in anxiety and depression symptom severity in autism. This is despite evidence that difficulties with emotion awareness relate to mental health symptoms in the general population (Mattila et al., 2008), including young children (Rieffe, Oosterveld, Miers, Meerum Terwogt, & Ly, 2008). Moreover, at least 20–50% of autistic individuals experience co-occurring anxiety and/or depression – far higher rates than those observed in the general population (Hollocks, Lerh, Magiati, Meiser-Stedman, & Brugha, 2019; Simonoff et al., 2008). Hence, research on mechanisms associated with co-occurring anxiety and depression in autism has been declared a research priority (Autistica, 2017; Pellicano, Dinsmore, & Charman, 2014), for which alexithymia may provide a tractable treatment target.

Some recent studies indicate that there may be associations between alexithymia and anxiety/depression symptoms in autism. For instance, Milosavljevic et al. (2016) showed that adolescents with autism and elevated alexithymia self-reported significantly higher concurrent anxiety than those with autism only. Similarly, alexithymia has been found to partially mediate associations between core autism traits and anxiety and depression symptoms in autistic young adults (Maisel et al., 2016; Morie, Jackson, Wei, Marc, & Dritschel, 2019). These findings imply that alexithymia may be a key contributing factor for mental health problems in autism, as in the general population.

Nevertheless, there is a considerable lack of longitudinal research examining the putative impact of alexithymia in autism (Kojima, 2012). Existing studies have been cross-sectional in nature and considered alexithymia as a unitary construct, when there may be subtle differences between its individual facets (e.g. difficulties in describing *v.* identifying emotions) and specific outcomes. As a result, the role of alexithymia (or its individual facets) in predicting the development of social-communication difficulties and/or mental health symptoms remains unclear.

For example, alexithymia may be a modifying factor that interacts with concurrent core autism traits and/or anxiety and depression symptoms, resulting in individual differences in outcome, or treatment response. Additionally, or alternatively, alexithymia may represent a precursor to later developing socio-emotional and mental health problems – potentially via difficulties with emotion perception/production (Brewer et al., 2016a; Trevisan & Birmingham, 2016), interoception (perception of internal bodily states; Garfinkel et al., 2016) and/or emotion regulation (Maisel *et al*. 2016; Morie *et al*. 2019). Finally, alexithymia may be a consequence of difficulties in understanding others' emotions (Carruthers, 2009), or act as a compensatory buffer against aversive internalising problems (Kojima, 2012; Mor & Winquist, 2002). Overall, establishing longitudinal relationships between potential precursors (e.g. alexithymia) and outcomes over time (e.g. social functioning, anxiety and depression) is essential for informing early and effective interventions and support in autism.

### The current study

Thus, our study first aimed to assess cross-sectional associations between alexithymia (including its individual facets), social-communication difficulties, anxiety and depression symptoms in a large, well-characterised cohort of autistic and non-autistic males and females, aged 12–30 years. Furthermore, we modelled longitudinal associations between earlier alexithymia and subsequent social-communication difficulties, anxiety and depression symptoms approximately 12–24 months later. Based on previous literature, we predicted that increasing alexithymia would be associated with elevated social-communication difficulties, anxiety and depression symptoms in the autism group, at a single timepoint and longitudinally.

## Methods

### Participants

The current study is based on data from the EU-AIMS Longitudinal European Autism Project (LEAP). A detailed description of the design and methodologies of LEAP are reported elsewhere (Loth et al., 2017). Briefly, LEAP employed a case-control design whereby males and females aged 6–30 years, with and without a formal diagnosis of autism spectrum disorder (ASD), were recruited simultaneously across seven European study sites. Subsequently, the cohorts were followed up longitudinally, after approximately 12–24 months. Participants were initially recruited via existing databases, schools and flyers; and those with mild intellectual disability (IQ ⩽ 75) were additionally recruited through clinic contacts or support groups. Although individuals with IQ ⩽ 75 were recruited to both ASD and comparison groups, we note that few met these criteria, partly due to the reasonably demanding nature of the full LEAP protocol and because some individuals believed to have mild intellectual disability based on an initial screener scored above 75 on standardised IQ measures (see Loth et al., 2017).

In total, 337 participants aged 12–30 years (ASD *N* = 179) with available alexithymia data were included in this study. Table 1 shows participants’ characteristics on entry to the study (timepoint one; T1). A total of 135 individuals (ASD *N* = 76) were able to be followed up approximately 12–24 months later (timepoint two; T2). T2 participant characteristics are shown in online Supplementary Table S2.

**Table 1.** Descriptives and group comparisons for participant characteristics and alexithymia at T1

(a) Demographic/clinical	ASD (T1)			Non-ASD (T1)			Group comparison		
	*N*	Median (IQR)	Range	*N*	Median (IQR)	Range	χ^2^ (_df, *N*_)	*p*	*ϕ*
Sex: males (females)	124 (55)	–	–	103 (55)	–	–	0.64 (_1,337_)	0.42	0.04
							*Z*	*p*	*r* effect size
Age (years)	179	19.57 (7.49)	12–30	158	19.11 (7.00)	12–30	0.35	0.73	0.02
Full-scale IQ	179	105.15 (20.09)	67–148	158	108.18 (18.50)	74–142	2.37	0.02*	0.13
SRS-2 (Self)	173	63.00 (13.00)	40–94	146	46.00 (7.00)	37–69	12.66	<0.001***	0.71
SRS-2 (Parent)	142	68.50 (18.00)	43–95	64	43.50 (7.00)	37–73	10.40	<0.001***	0.72
RBS-R	147	11.00 (14.50)	0–90	64	0.00 (1.00)	0–13	9.89	<0.001***	0.68
Beck's Anxiety	168	0.16^a^ (1.32)	−1.28 to 4.65^a^	150	−0.60^a^ (0.72)	−1.28 to 1.88^a^	7.35	<0.001***	0.41
Beck's Depression	168	0.05^a^ (1.37)	−0.97 to 4.15^a^	149	−0.65^a^ (0.55)	−1.08 to 2.16^a^	8.00	<0.001***	0.45
(b) Alexithymia	ASD (T1)			Non-ASD (T1)			Group comparison		
	*N*	Median (IQR)	Range	*N*	Median (IQR)	Range	*Z*	*p*	*r* effect size
TAS total	179	52.00 (17.00)	24–88	158	40.00 (14.75)	20–69	8.85	<0.001***	0.48
TAS identify	179	15.00 (10.00)	7–35	158	9.00 (6.00)	7–30	8.05	<0.001***	0.44
TAS describe	179	16.00 (7.00)	5–25	158	10.00 (5.00)	5–24	8.21	<0.001***	0.45
TAS external	179	22.00 (6.00)	8–40	158	20.00 (7.00)	8–35	4.04	<0.001***	0.22

SRS-2, Social Responsiveness Scale-Second Edition; RBS-R, Repetitive Behaviour Scale-Revised; TAS, Toronto Alexithymia Scale; IQR, interquartile range; *Z*, statistic for Mann–Whitney comparison; χ^2^_(df, *N*)_, χ^2^ test (degrees of freedom, number of participants); *r*, effect size (*Z*/√*N*).

a Scores *z*-transformed for comparability between youth and adult versions.

***p < 0.004 (significant after Bonferroni correction; *p* = 0.05/12).

On entry to the study, there were no significant differences between autistic and comparison individuals in age (*p* = 0.73, *r* effect size = 0.02) nor sex (*p* = 0.42, *ϕ* = 0.04). Autistic individuals had marginally lower average IQ than comparison individuals (*p* = 0.02, *r* = 0.13), although median scores were fairly comparable between groups (ASD median = 105.15, IQR = 20.09; comparison median = 108.18, IQR = 18.50). Autistic individuals had significantly more core autism traits (T1: *p* < 0.001; *r* ⩾ 0.68; T2: *p* < 0.001; *r* ⩾ 0.54) and higher mental health symptom severity (T1: *p* < 0.001; *r* ⩾ 0.41; T2: *p* < 0.001; *r* ⩾ 0.34) than comparison individuals across all measures, at both timepoints.

Participants who were able to be followed up at T2 did not significantly differ from non-returners on baseline age/sex (*p* ⩾ 0.14), alexithymia (*p* = 0.22), social-communication difficulties (*p* ⩾ 0.12), nor depression symptoms (*p* = 0.36). Returners had slightly higher IQ (*p* = 0.01) and lower anxiety (*p* = 0.05) and repetitive behaviours than non-returners (*p* = 0.02). Reasons for loss to follow-up included: participants returning at T2 but not completing the alexithymia measure; inability to make contact with participants; and personal circumstances that made participants unable to visit at T2.

### Materials and procedures

#### Alexithymia

See online Supplementary Table S1 for a summary of all measures. To index alexithymia, we administered the 20-item self-report Toronto Alexithymia Scale (TAS-20; Bagby et al., 1994), which has been previously validated for use in autism (Berthoz & Hill, 2005). The TAS-20 results in a total score and three subscales: difficulties in identifying feelings (*I am often puzzled by sensations in my body*), difficulties in describing feelings (*It is difficult for me to find the right words for my feelings*) and externally oriented thinking (*I prefer to analyse my problems rather than just describe them*). As evident from these example items, one's awareness of their difficulties with emotions seems somewhat distinct from one's awareness that they are experiencing a positive or adverse emotional state.

Higher scores indicate higher alexithymia, with scores ⩾61 indicating ‘severe’ (i.e. clinically relevant) alexithymia (Parker, Taylor, & Bagby, 2003). Alexithymia was weakly associated with verbal IQ – significant in the whole sample (*r*_s_ = −0.24, *p* < 0.001) and marginal in the ASD group (*r*_s_ = −0.16, *p* = 0.03), suggesting results were not wholly explained by difficulties in completing the TAS due to individual differences in verbal ability.

#### Core autism traits

The clinical characteristics of the full LEAP cohort are reported elsewhere (Charman et al., 2017). In the current study, social-communication difficulties were measured using self- and parent-report versions of the Social Responsiveness Scale-2nd Edition (SRS-2; Constantino and Gruber, 2012). Higher scores (sex-specific *T*-norms) indicate more difficulties. Repetitive behaviours were measured using the parent-report Repetitive Behaviour Scale-Revised (RBS-R; Lam and Aman, 2007), with higher scores indicating more repetitive behaviours.

#### Anxiety/depression symptoms

Beck's Anxiety and Depression Inventories (Beck, Beck, Jolly, & Steer, 2005; Beck, Epstein, Brown, & Steer, 1988; Beck, Steer, & Brown, 2001) were used to capture self-reported anxiety and depression symptoms at two timepoints, ~12–24 months apart. Beck's Inventories were chosen on the basis of their fast administration time (reducing longitudinal participant burden) and ability to accurately screen for mental health problems in general population and autism samples (Cassidy, Bradley, Bowen, Wigham, & Rodgers, 2018; Leyfer, Ruberg, & Woodruff-Borden, 2006). We administered a youth-version for ages 12–17 years and an adult-version for ages 18–30 years. Therefore, scores were *z*-transformed for comparability, with higher scores indicating more severe symptoms.

### Statistical analyses

Analyses were performed using RStudio^®^. As some data violated the normality assumption, non-parametric analyses were used for group comparisons (Mann–Whitney) and correlations (Spearman's *r*_s_). For group comparisons, effect size *r* (calculated as Z/√N) is reported, with 0.10 denoting a small effect, 0.30 a moderate effect and 0.50 a large effect. Bonferroni correction was applied throughout. See Supplementary Table S3 for correlations between all T1 and T2 measures.

To investigate cross-sectional associations between alexithymia, and clinical features of social-communication difficulties, anxiety and depression symptoms, we first compared those who did *v.* did not meet cut-off (⩾61) for clinically relevant alexithymia – an approach that would also capture potentially non-linear relationships. Next, we investigated both cross-sectional and longitudinal relationships between alexithymia and clinical features in greater depth using linear (‘lm’) regression models. Dependent variables were, respectively, SRS-2-rated social-communication difficulties and Beck-rated anxiety/depression symptom. Independent variables across all models were age, IQ, sex and alexithymia (total/subscale scores).

## Results

### Alexithymia in the current sample

Autistic individuals reported, on average, significantly higher total alexithymia than comparison individuals, with a moderate effect size (*p* < 0.001, *r* = 0.48). Autistic individuals scored significantly higher than comparison individuals across TAS-20 subscales, particularly difficulties in identifying (*p* < 0.001, *r* = 0.44) and describing emotion (*p* < 0.001, *r* = 0.45; Table 1).

**Fig. 1.**
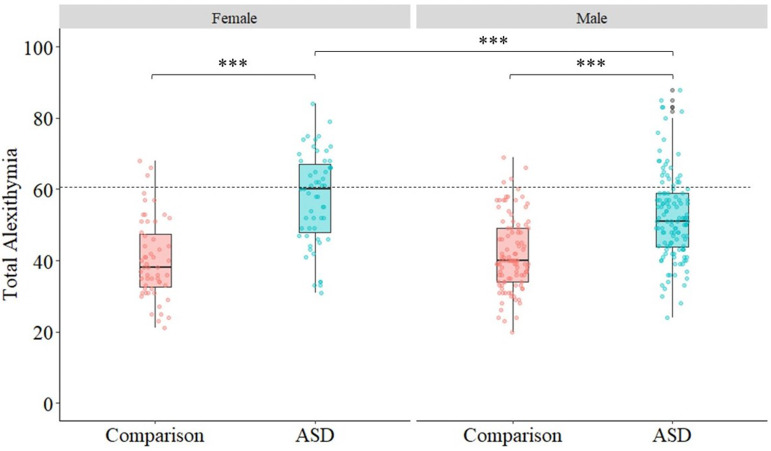
Box plots showing median and IQR of total alexithymia in ASD and comparison groups, split by sex. Individual data plots are overlaid to demonstrate the variability of scores in both groups. Cut-off for severe alexithymia (⩾61) is indicated by the dashed line.

As demonstrated in Fig. 1, 29.1% (*N* = 52) of autistic individuals met cut-off for clinically relevant alexithymia (score ⩾61; Parker et al., 2003), compared to 4.4% (*N* = 7) of comparison individuals. Autistic females were over-represented in the group who met cut-off (*p* = 0.001, χ^2^_(1179)_ = 11.55, *ϕ* = 0.27). A total of 47.3% (*N* = 26) of autistic females met cut-off, compared to 21.0% (*N* = 26) of males. Autistic females in our sample also scored higher than males for self-reported social-communication difficulties (*p* = 0.01, *r* = 0.20). Conversely, there was no significant sex difference in alexithymia in the comparison group (*p* = 0.96, χ^2^_(1158)_ = 0.003, *ϕ* = 0.04).

### T1 associations between alexithymia and social-communication difficulties

First, we used a categorical approach to confirm that individuals with clinically relevant alexithymia scored higher for social-communication difficulties than those with lower levels of alexithymia (see online Supplementary Table S4). Overall, individuals who met cut-off for severe alexithymia had significantly higher SRS-2 self-reported social-communication difficulties (*p* < 0.001, *r* = 0.55) and, to a lesser degree, parent-reported social-communication difficulties (*p* < 0.001, *r* = 0.33) than those with lower levels of alexithymia. Similarly, in the ASD group, individuals with severe alexithymia scored high for SRS-2 self- (*p* < 0.001, *r* = 0.61) and parent-reported social-communication difficulties (*p* = 0.01, *r* = 0.22).

Next, we considered dimensional relationships between alexithymia (sub)scale scores and social-communication difficulties, controlling for potential confounds of age, IQ, sex and concurrent repetitive behaviours. In the whole sample, alexithymia remained significantly associated with SRS-2 self-reported social-communication difficulties, across all TAS-20 (sub)scales, with the strongest effect size for difficulties in describing feelings: total (*p* < 0.001, *β* = 0.62, 95% confidence interval (CI) 0.52–0.69), identify (*p* < 0.001, *β* = 0.53, 95% CI 0.40–0.59), describe (*p* < 0.001, *β* = 0.57, 95% CI 0.46–0.64) and external (*p* < 0.001, *β* = 0.28, 95% CI 0.16–0.38). Difficulty in describing feelings was also significantly related to SRS-2 parent-reported social-communication difficulties (*p* = 0.001, *β* = 0.19, 95% CI 0.08–0.29).

In the ASD group, alexithymia remained significantly associated with SRS-2 self- (but not parent-) reported social-communication difficulties, across all TAS-20 (sub)scales, with the strongest effect size for difficulties in describing feelings: total (*p* < 0.001, *β* = 0.64, 95% CI 0.51–0.73), identify (*p* < 0.001, *β* = 0.52, 95% CI 0.37–0.62), describe (*p* < 0.001, *β* = 0.57, 95% CI 0.44–0.67) and external (*p* = 0.001, *β* = 0.25, 95% CI 0.10–0.38).

### T1 associations between alexithymia and anxiety/depression symptoms

Similar to the findings reported above, individuals who met cut-off for severe alexithymia had significantly higher T1 Beck-rated anxiety and depression symptom severity, as compared to those with lower levels of alexithymia – in the whole sample (*p* < 0.001, *r* ⩾ 0.42) and ASD group (*p* < 0.001, *r* ⩾ 0.46; online Supplementary Table S4).

Once again, we assessed dimensional relationships between alexithymia and anxiety/depression symptoms, controlling for potential confounds of age, IQ, sex and core autism traits. Based on findings from simple correlations (online Supplementary Table S3), we ran linear regression models for associations between all alexithymia (sub)scale scores, except for externally oriented thinking.

As shown in Table 2, difficulties in identifying feelings were significantly associated with anxiety symptom severity in the whole sample (*p* < 0.001, *β* = 0.54, 95% CI 0.41–0.69). Of note, when depression symptoms were also controlled for, the relationship between difficulties in identifying feelings and anxiety remained (*p* < 0.001, *β* = 0.28, 95% CI 0.15–0.40). The same pattern of effects was identified in the ASD group. Difficulties in identifying feelings accounted for significant variance in anxiety symptoms (*p* < 0.001, *β* = 0.54, 95% CI 0.41–0.77), even after controlling for depression (*p* = 0.001, *β* = 0.27, 95% CI 0.12–0.46).

**Table 2.** Regression coefficients and model fit statistics for T1 Beck-rated anxiety and depression symptom severity, regressed onto alexithymia scores, controlling for demographic factors and core autism traits in the: (a) whole sample; (b) ASD group

			(a) All		(b) ASD	
			Beck's Anxiety (T1)	Beck's Depression (T1)	Beck's Anxiety (T1)	Beck's Depression (T1)
			*β* (95% CI)	*β* (95% CI)	*β* (95% CI)	*β* (95% CI)
Model 1	Demographic	Age	−0.08 (−0.22 to 0.04)	−0.15 (−0.29 to −0.03)*	−0.05 (−0.23 to 0.11)	−0.15 (−0.34 to 0.01)
		IQ	−0.10 (−0.22 to 0.01)	−0.02 (−0.14 to 0.10)	−0.18 (−0.35 to −0.04)*	−0.07 (−0.24 to 0.09)
		Sex	−0.12 (−0.26 to −0.01)*	−0.13 (−0.27 to −0.02)*	−0.08 (−0.28 to 0.08)	−0.11 (−0.31 to 0.05)
	Autism traits	SRS-2	0.45 (0.27–0.70)***	0.53 (0.35–0.78)***	0.39 (0.19–0.71)***	0.38 (0.16–0.71)**
		RBS-R	0.03 (−0.11 to 0.18)	−0.09 (−0.24 to 0.05)	0.06 (−0.12 to 0.25)	−0.10 (−0.30 to 0.08)
	Alexithymia	Total	0.21 (0.04–0.41)*	0.20 (0.02–0.39)*	0.18 (−0.04 to 0.44)	0.22 (−0.01 to 0.49)
Model fit (*R*^2^_adj_)			*F*_(6,191)_ = 23.58, *p* < 0.001***, *η*_p_^2^ = 0.64 (40.8%)	*F*_(6,189)_ = 21.34, *p* < 0.001***, *η*_p_^2^ = 0.63 (38.5%)	*F*_(6,130)_ = 12.64, *p* < 0.001***, *η*_p_^2^ = 0.61 (33.9%)	*F*_(6,128)_ = 9.58, *p* < 0.001, *η*_p_^2^ = 0.59 (27.8%)
Model 2	Demographic	Age	−0.15 (−0.28 to −0.05)**	−0.21 (-0.35 to −0.11)***	−0.13 (−0.30 to 0.01)	−0.22 (−0.41 to −0.08)**
		IQ	−0.09 (−0.19 to 0.01)	−0.01 (−0.12 to 0.09)	−0.16 (−0.32 to −0.04)**	−0.06 (−0.21 to 0.08)
		Sex	−0.05 (−0.16 to 0.06)	−0.07 (−0.19 to 0.04)	−0.02 (−0.18 to 0.14)	−0.04 (−0.22 to 0.11)
	Autism traits	SRS-2	0.26 (0.11–0.44)***	0.37 (0.22–0.57)***	0.21 (0.05–0.44)**	0.24 (0.06–0.49)**
		RBS-R	0.04 (−0.09 to 0.17)	−0.08 (−0.23 to 0.04)	0.05 (−0.10 to 0.22)	−0.10 (−0.29 to 0.05)
	Alexithymia	Identify	0.54 (0.41–0.69)***	0.48 (0.34–0.63)***	0.54 (0.41–0.77)***	0.52 (0.36–0.74)***
Model fit (*R*^2^_adj_)			*F*_(6,191)_ = 39.32, *p* < 0.001***, *η*_p_^2^ = 0.69 (53.9%)	*F*_(6,189)_ = 31.96, *p* < 0.001***, *η*_p_^2^ = 0.67 (48.8%)	*F*_(6,130)_ = 22.70, *p* < 0.001***, *η*_p_^2^ = 0.67 (48.9%)	*F*_(6,128)_ = 16.49, *p* < 0.001***, *η*_p_^2^ = 0.64 (41.0%)
Model 3	Demographic	Age	−0.10 (−0.24 to 0.03)	−0.16 (−0.31 to −0.04)**	−0.06 (−0.24 to 0.11)	−0.15 (−0.35 to 0.01)
		IQ	−0.12 (−0.24 to −0.01)*	−0.04 (−0.16 to 0.07)	−0.20 (−0.37 to −0.06)**	−0.10 (−0.27 to 0.06)
		Sex	−0.12 (−0.27 to −0.01)*	−0.13 (−0.28 to −0.02)*	−0.08 (−0.28 to 0.08)	−0.11 (−0.31 to 0.06)
	Autism traits	SRS-2	0.63 (0.48–0.88)***	0.71 (0.56–0.97)***	0.60 (0.44–0.93)***	0.60 (0.43–0.95)***
		RBS-R	0.02 (−0.12 to 0.17)	−0.10 (−0.26 to 0.04)	0.05 (−0.13 to 0.24)	−0.10 (−0.30 to 0.07)
	Alexithymia	Describe	−0.01 (−0.18 to 0.16)	−0.03 (−0.21 to 0.14)	−0.10 (−0.34 to 0.10)	−0.08 (−0.32 to 0.14)
Model fit (*R*^2^_adj_)			*F*_(6,191)_ = 21.98, *p* < 0.001***, *η*_p_^2^ = 0.63 (39.0%)	*F*_(6,189)_ = 20.07, *p* < 0.001***, *η*_p_^2^ = 0.62 (37.0%)	*F*_(6,130)_ = 12.21, *p* < 0.001***, *η*_p_^2^ = 0.61 (33.1%)	*F*_(6,128)_ = 8.86, *p* < 0.001***, *η*_p_^2^ = 0.59 (26.0%)

T1, timepoint 1; SRS-2, Social Responsiveness Scale-Second Edition (self-report); RBS-R, Repetitive Behaviour Scale-Revised; *β* (95% CI), regression coefficient with 95% confidence intervals.

*p < 0.05; **p < 0.01; ***p < 0.001 (significant after Bonferroni correction; *p* = 0.05/72).

In contrast, although difficulties in identifying feelings were also significantly associated with depression in the whole sample (*p* < 0.001, *β* = 0.48, 95% CI 0.34–0.63), this association fell to the marginal level for significance after controlling for anxiety (*p* = 0.04, *β* = 0.14, 95% CI 0.01–0.28). Similarly, in the ASD group, difficulties in identifying feelings were significantly associated with depression symptoms (*p* < 0.001, *β* = 0.52, 95% CI 0.36–0.74), but marginally after controlling for anxiety (*p* = 0.03, *β* = 0.19, 95% CI 0.02–0.38).

Post-hoc analysis using the ‘mediate’ function (10 000 iterations) showed that anxiety partly mediated associations between difficulties in identifying feelings and depression. *a* denotes the path from difficulty in identifying feelings to anxiety; *b* is the path from anxiety to depression symptoms and *c*′ is the direct path from difficulty in identifying feelings to depression. Although the direct path from difficulty in identifying feelings to depression remained (whole sample *c*′ = 0.04, s.e. = 0.01, *p* < 0.001; ASD *c*′ = 0.04, s.e. = 0.01, *p* < 0.001), anxiety was a significant, partial mediator of this relationship (whole sample *a* = 0.10, s.e. = 0.01, *p* < 0.001; *b* = 0.62, s.e. = 0.04, *p* < 0.001; ASD *a* = 0.10, s.e. = 0.01, *p* < 0.001; *b* = 0.58, s.e. = 0.06, *p* < 0.001).

### Longitudinal associations between alexithymia, social-communication difficulties, anxiety and depression symptoms

#### Stability in measures over time

For those who participated at both timepoints, paired difference tests showed that, at the group level, alexithymia severity did not significantly change over time (*p* = 0.60, *r* = 0.05). Similarly, there was no significant change in self-reported social-communication difficulties (*p* = 0.17, *r* = 0.18), anxiety (*p* = 0.08, *r* = 0.15), nor depression symptoms (*p* = 0.10, *r* = 0.14).

**Fig. 2.**
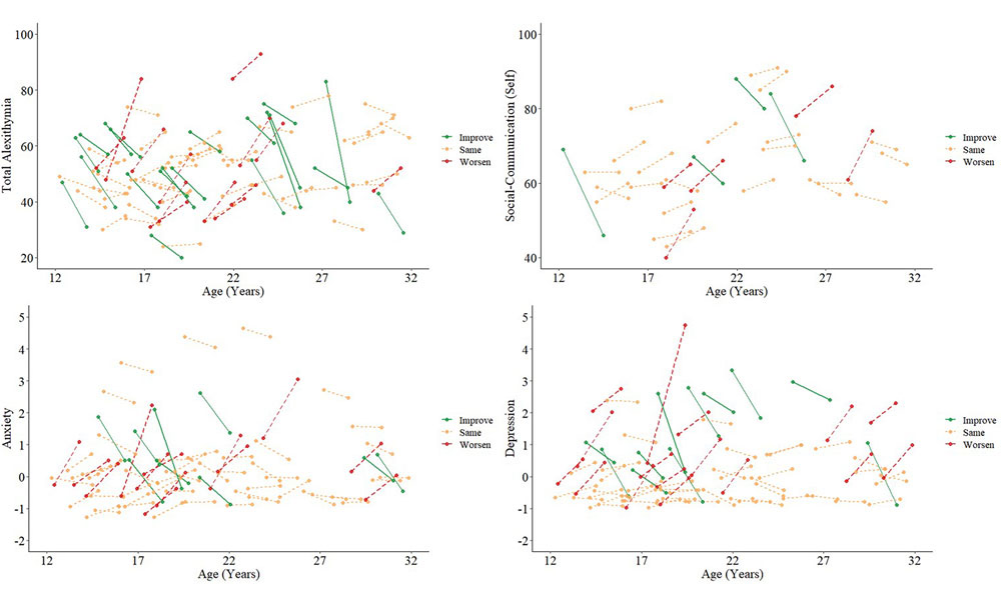
Individual trajectories of alexithymia, self-reported social-communication difficulties, anxiety and depression from T1 to T2 for autistic individuals in our cohort. Few individuals experienced ‘minimally clinically important’ change (>0.5 s.d. from baseline score): total alexithymia *N* = 13 ‘worsen’, *N* = 20 ‘improve’, *N* = 43 ‘same’; self-reported social-communication difficulties *N* = 5 ‘worsen’, *N* = 4 ‘improve’, *N* = 22 ‘same’; anxiety *N* = 13 ‘worsen’, *N* = 9 ‘improve’, *N* = 48 ‘same’; depression *N* = 16 ‘worsen’, *N* = 11 ‘improve’, *N* = 44 ‘same’.

These effects were consistent when considering the ASD group only (alexithymia: *p* = 0.47, *r* = 0.08; social-communication: *p* = 0.22, *r* = 0.22; anxiety: *p* = 0.48, *r* = 0.08; depression: *p* = 0.20, *r* = 0.15; see Table 1 and online Supplementary Table S2 for descriptives). At the individual level, fewer than half of autistic participants exhibited a minimally clinically important change of >0.5 s.d. on any variable, between timepoints (see Norman, Sloan, and Wyrwich, 2003). Individual longitudinal trajectories for alexithymia, social-communication difficulties, anxiety and depression are shown in Fig. 2.

#### Social-communication difficulties

**Fig. 3.**
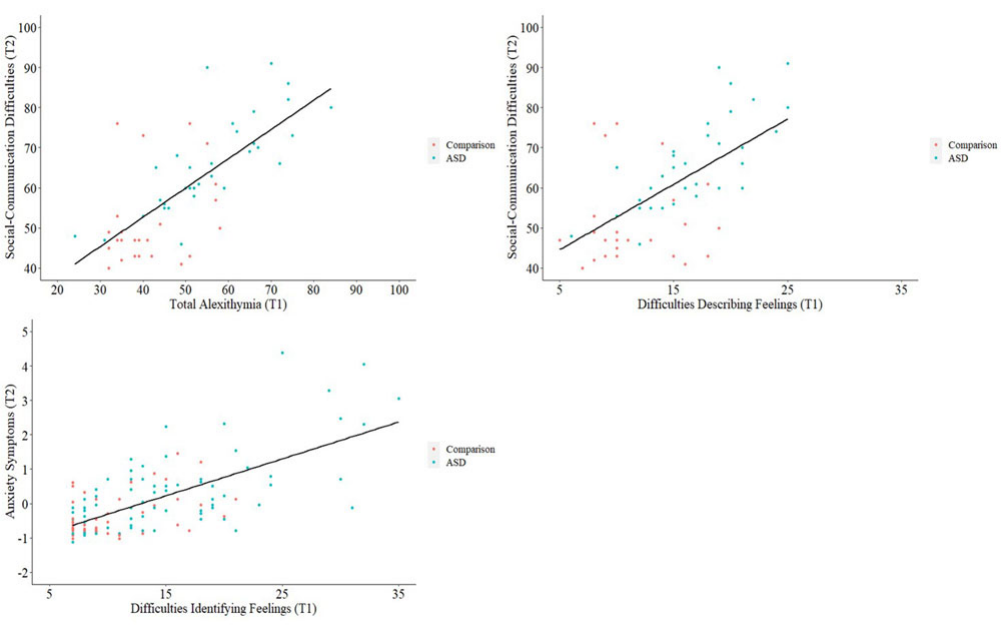
Scatterplots with regression lines fitted, showing longitudinal relationships between T1 alexithymia and T2 self-reported social-communication difficulties and anxiety symptoms.

To ascertain whether earlier alexithymia was associated with later self-reported social-communication difficulties, anxiety and depression symptoms, over ~12–24 months, we conducted longitudinal regression analyses (Fig. 3).

In the whole sample, no associations between alexithymia and social-communication difficulties were maintained longitudinally. Conversely, in the ASD group, T1 total alexithymia remained marginally associated with T2 self-reported social-communication difficulties, after controlling for age, IQ, sex, repetitive behaviours and T1 self-reported social-communication difficulties (*p* = 0.02, *β* = 0.52, 95% CI 0.09–0.92). The same was true for the association between T1 difficulties in describing feelings and T2 self-reported social-communication difficulties (*p* = 0.02, *β* = 0.43, 95% CI 0.07–0.82), although we caution that only *N* = 22 autistic individuals had sufficient longitudinal data. Overall both models were significant and accounted for over 59.0% of the variance in T2 self-reported social-communication difficulties in the ASD group (total *F*_(6,22)_ = 8.01, *p* < 0.001, *η*_p_^2^ = 0.76, *R*^2^_adj_ = 60.0%; describe *F*_(6,22)_ = 7.88, *p* < 0.001, *η*_p_^2^ = 0.76; *R*^2^_adj_ = 59.6%).

#### Anxiety/depression symptoms

T1 difficulty in identifying feelings remained associated with T2 anxiety symptoms in individuals with sufficient longitudinal data from both the whole sample (*N* = 76; *p* = 0.002, *β* = 0.32, 95% CI 0.12–0.52) and ASD group (*N* = 51; *p* = 0.01, *β* = 0.31, 95% CI 0.08–0.62), after accounting for demographic factors, core autism traits and T1 anxiety/depression symptoms. The overall model for this association was significant and accounted for over 69.0% of the variance in T2 anxiety symptom severity (whole sample *F*_(8,76)_ = 26.43, *p* < 0.001, *η*_p_^2^ = 0.79 *R*^2^_adj_ = 70.8%; ASD *F*_(8,51)_ = 17.86, *p* < 0.001, *η*_p_^2^ = 0.79 *R*^2^_adj_ = 69.6%).

No other longitudinal models showed significant associations between T1 alexithymia and T2 anxiety/depression symptoms, after controlling for other factors. Nevertheless, in simple correlations, T1 difficulties in identifying feelings remained associated with T2 depression (whole sample: *r*_s_ = 0.47, *p* < 0.001; ASD: *r*_s_ = 0.51, *p* < 0.001; Supplementary Table S3).

## Discussion

### Alexithymia and social-communication difficulties in autism

To our best knowledge, this is the most comprehensive study to date to assess cross-sectional – and the first to assess longitudinal – associations between alexithymia, social-communication difficulties, anxiety and depression symptoms in autistic males and females, during adolescence and early adulthood. First, we found that all facets of alexithymia (total severity, difficulties in identifying/describing feelings and externally oriented thinking) were significantly associated with concurrent self- (but not parent-) reported social-communication difficulties in autism. The strongest associations were between total alexithymia/difficulties in describing feelings and self-reported social-communication difficulties. Furthermore, associations between both total alexithymia and difficulties in describing feelings with self-reported social-communication difficulties were maintained longitudinally (albeit in a smaller subsample for whom data were available), and this was true even after controlling for age, IQ, sex, repetitive behaviours and earlier T1 social-communication difficulties.

This finding suggests that autistic individuals with lower emotional awareness, particularly those who find it challenging to describe their emotions, also perceive themselves to experience more interpersonal difficulties – although these difficulties may not be observed by others. This corresponds to previous findings from individuals with high alexithymia without autism (and analyses including the comparison group in this study), for whom difficulties with describing feelings are also the facet of alexithymia most strongly associated with interpersonal problems (Spitzer et al., 2005).

One possible explanation for the observed associations between alexithymia and social-communication difficulties is that difficulties with emotion recognition and production disrupt everyday social interactions (Bothe, Palermo, Rhodes, Burton, & Jeffery, 2019; Trevisan & Birmingham, 2016). For instance, previous studies suggest some autistic individuals find it challenging to interpret others' facial emotion expressions; and non-autistic individuals experience difficulties in interpreting the expressions of autistic people (Brewer et al., 2016a; Loth, Ahmad, Watson, Duff, & Duchaine, 2018). Furthermore, given that difficulty in describing feelings was the alexithymia subscale most strongly related to social-communication difficulties, it is likely that broader (emotional) language impairments may be implicated (Lartseva, Dijkstra, & Buitelaar, 2015). Indeed, in the current study, verbal ability was significantly (although weakly) associated with alexithymia in both the whole sample and autism group. In the wider literature, ‘acquired’ alexithymia has been reported following traumatic brain injury affecting language regions (Hobson et al., 2018) and language delay/reduced depth of verbal expression are common in autism – observed from early development (Baird et al., 2006; Rieffe et al., 2007).

Nevertheless, since alexithymia was associated with self- but not parent-reported social difficulties in the autism group, it is also possible that some autistic individuals are highly aware of the impact of their emotional understanding on interpersonal relationships and experience low confidence in their social abilities. For example, difficulties in recognising the emotions and thoughts of others may lead to real-world negative social encounters or ‘catastrophising’ negative beliefs (e.g. ‘they must be upset with me’), which increase self-consciousness in future social interactions (Capriola, Maddox, & White, 2017; Spain, Sin, & Freeman, 2016; van Roekel, Scholte, & Didden, 2010). Therefore, interventions for improving social communication in autism should not only focus on social skills, but also on emotional awareness. This may have wider benefits for improving subjective perceptions of social competence, reducing social anxieties and increasing confidence in social settings.

### Difficulties in identifying feelings and anxiety/depression symptoms in autism

In addition, difficulties in identifying feelings were significantly and robustly associated with anxiety symptoms in autistic adolescents and adults – even after controlling for demographic factors, core autism traits and concurrent depression. Moreover, using longitudinal data, we ascertained that earlier difficulties in identifying feelings remained associated with anxiety symptoms approximately 12–24 months later, also after accounting for earlier T1 anxiety. Since alexithymia and anxiety were stable over time in over half of autistic individuals in this sample, findings suggest that the ability to accurately interpret one's own emotional state has a significant and persistent impact on mental health outcomes in autism. Overall, our findings lend support to the suggestion that, as progress is made in developing and modifying interventions for autistic people, the potentially primary role of emotional awareness must be considered (Conner et al., 2019).

The reported findings have wider implications for informing early intervention strategies to improve mental health outcomes in autism. Currently, cognitive behavioural therapy (CBT) is the most widely supported intervention for anxiety in autism (White et al., 2018). Similar to many psychological therapies, this approach requires individuals to reflect on and discuss emotions and their antecedents. Yet, paradoxically, autistic individuals with particularly low emotional awareness are the most likely to be referred for CBT (likely due to associated mental health problems; Roberts-Collins, Mahoney-Davies, Russell, Booth, and Loades, 2018). This raises several questions around the long-term effectiveness of current treatment approaches and highlights the stark need for additional research to develop and evaluate treatment modifications for managing mental health symptoms in autism (Walters, Loades, & Russell, 2016). Notably, emotion recognition training is included in current guidance from the UK National Institute for Health and Care Excellence on the adaptation of CBT for autism (National Institute of Health & Care Excellence, 2013), for which this study provides empirical support.

A key direction for future research is, therefore, to assess whether incorporating techniques specifically targeting emotional awareness could improve therapeutic outcomes for autistic people. For instance, psychological interventions that specifically target, rather than passively measure, alexithymia have been found to be successful in reducing alexithymic traits and improving emotion regulation skills in neurotypical populations (Cameron, Ogrodniczuk, & Hadjipavlou, 2014). Such methods have tended to focus on psychoeducation and stress-management. However, the efficacy of these techniques for improving outcome in autism, and other neurodevelopmental/neuropsychiatric conditions characterised by high rates of alexithymia, is yet to be confirmed (Cooper, Loades, & Russell, 2018).

Another outstanding issue for future research is to establish the mechanistic processes that connect emotional awareness and mental health outcomes (e.g. anxiety) over time. Emotion regulation difficulties are one candidate underpinning mechanism for mental health gaining increasing attention in the autism literature (Cai, Richdale, Uljarević, Dissanayake, & Samson, 2018), with emotion awareness argued to be the first and most fundamental aspect of successful emotion regulation (Gross, 2015). For instance, if the accurate identification of one's current emotion is impeded, this may hinder the implementation of adaptive emotion regulation strategies, including cognitive reappraisal (reinterpreting the emotion) and situation selection/modification (adjusting the environment; Gross and Thompson, 2007).

Adaptive emotion regulation may also be impeded by broader difficulties in interpreting internal bodily states (i.e. interoception) that indicate heightened arousal or anxiety, such as elevated heart and breathing rate (Garfinkel, Eccles, & Critchley, 2015; Müller et al., 2015). Of note, the Beck's Anxiety Inventory indexes several physiological anxiety symptoms (heart/breathing rate and trembling) and some previous studies suggest that alexithymia is related to general interoceptive impairments, across affective and non-affective domains (Brewer, Cook, & Bird, 2016b; Murphy, Catmur, & Bird, 2018). Furthermore, alongside alexithymia, autistic individuals have been shown to experience elevated interoception and emotion regulation difficulties – engaging in fewer ‘voluntary’ (e.g. cognitive reappraisal – reinterpreting emotional stimuli) and more ‘involuntary’ regulation strategies (e.g. rumination and arousal) than neurotypical individuals (Gross, 2015; Mazefsky, Borue, Day, & Minshew, 2014; Samson, Hardan, Lee, Phillips, & Gross, 2015). Thus, difficulties with emotional, and broader internal bodily, awareness may compromise ‘downstream’ emotion regulation processes, increasing vulnerability for mental health problems such as anxiety.

Finally, we note that while relationships between difficulties in identifying feelings and anxiety symptoms were robust, we identified a more complex pattern of results for depression. Difficulties in identifying feelings and depression were associated cross-sectionally, although associations fell to the marginal level for significance after also controlling for anxiety. In addition, after accounting for other factors, earlier difficulties in identifying feelings were not longitudinally associated with depression symptoms 12–24 months later, in the whole sample, nor the autism group. This may suggest that associations between alexithymia and depression symptoms are explained by wider shared factors, such as co-occurring anxiety (Milosavljevic et al., 2016), or situational factors such as social withdrawal and loneliness (Hedley, Uljarević, Foley, Richdale, & Trollor, 2018). In support of this suggestion, a post-hoc mediation analysis showed that anxiety partly mediated cross-sectional associations between difficulties in identifying feelings and depression.

### Strengths and limitations

A major strength of our study is the inclusion of a heterogeneous, well-characterised sample of autistic males and females, aged 12–30 years. By including females, we were able to ascertain that significantly more diagnosed autistic females than males report high alexithymia. This implies that observed sex differences in rates of co-occurring emotional symptoms in autism (i.e. higher rates of anxiety/depression in autistic females; Mandy et al., 2012) may be explained by sex differences in their underpinning mechanisms. Furthermore, to our best knowledge, this is the first study to report longitudinal associations between alexithymia and social-communication difficulties, anxiety and depression symptoms in autism. We were thus able to confirm that alexithymia in our cohort was generally stable over a period of approximately 12–24 months, supporting the conceptualisation of alexithymia as a trait, rather than state (Salminen, Saarijärvi, Ääirelä, & Tamminen, 1994). However, we also acknowledge several limitations.

First, although we report associations between alexithymia and anxiety/depression symptoms for the whole sample (including the comparison group), in addition to the autism group, these must be interpreted with some caution. This is because clinically relevant mental health symptoms were screened out in the comparison (but not ASD) group on recruitment. We chose to report findings including our comparison group to address questions pertaining to specificity. For instance, associations between alexithymia and concurrent social-communication difficulties, as well as between alexithymia and concurrent/later anxiety symptoms were consistent between the whole sample and autism group. This adds to literature suggesting that the role of alexithymia in relation to mental health outcomes is not specific to a single neurodevelopmental/neuropsychiatric condition (e.g. autism), but instead a broader, diagnostically cross-cutting mechanism for mental health (National Institute of Mental Health, 2019). Furthermore, our findings imply that there are likely significant ‘commonalities’ in the nature of underpinning risk factors for mental health symptoms between autistic and neurotypical individuals, however they may differ in frequency/intensity.

Second, due to the lack of availability of self-report alexithymia measures for children and individuals with intellectual disability (Griffin, Lombardo, & Auyeung, 2016; Loas, Braun, Delhaye, & Linkowski, 2017), we were only able to include autistic individuals aged ⩾12 years, with IQ ⩾ 67. This prevented us from assessing whether associations between alexithymia and anxiety and depression symptoms were consistent across developmental stage and level and from generalising findings beyond the age range of 12–30 years. Indeed, a longitudinal period of 12–24 months is a reasonably short window for capturing traits that may have been established earlier in development. Therefore, a prospective longitudinal approach will be beneficial for further elucidating the mechanistic pathways linking alexithymia and outcomes in autism. In relation to this point, we were unable to assess the role of alexithymia in predicting *change* in social-communication difficulties, anxiety and depression symptoms between timepoints, due to the small number of participants who significantly changed on these features within the study period. It is still notable that we were able to include autistic individuals from age 12, since early adolescence is the developmental stage at which anxiety and depression symptoms most commonly emerge for both autistic and non-autistic individuals (Kessler et al., 2005; Simonoff et al., 2008). Nevertheless, the development and validation of tools for indexing alexithymia in under-represented groups is paramount (see Gaigg, Cornell, and Bird, 2016).

## Conclusions

Alexithymia, and particularly difficulty in describing feelings, is significantly associated with greater self-reported social-communication difficulties in autism, both cross-sectionally and longitudinally. Furthermore, difficulty in identifying feelings is related to anxiety symptom severity, including over a longitudinal period of at least 12–24 months. Taken together, these findings show that the influence of alexithymia on social functioning and anxiety is both substantial and persistent for autistic people – and possibly for autistic girls and women in particular. This has notable implications for intervention, indicating that psychological therapies targeting emotional awareness may improve later outcomes for some individuals.

## Supplementary material

For supplementary material accompanying this paper visit https://doi.org/10.1017/S0033291720003244. click here to view supplementary material
