# Supplementary material for: Alexithymia in autism: cross-sectional and longitudinal associations with social-communication difficulties, anxiety and depression symptoms
Source: Psychol Med. 2020 Oct 8;52(8):1458–70. doi: 10.1017/S0033291720003244 (PMC9226426; doi:10.1017/S0033291720003244)
Supplement: Supplementary file 1 [file S0033291720003244sup.zip › S0033291720003244sup001.docx]

Supplementary Table 1.

Summary of measures included in the current study, including timepoint of assessment and informant.

| Construct | Measure | Timepoint | Age Range | |
| --- | --- | --- | --- | --- |
|  |  |  | **Adol. (12-17-years)** | **Adults (18-30-years)** |
| Alexithymia | **20-item Toronto Alexithymia Scale** | 1 & 2 | S | S |
| Autism traits | **Social Responsiveness Scale - Second Edition** | 1 & 2 | S & P | S & P |
|  | **Repetitive Behaviour Scale - Revised** | 1 & 2 | P | P |
| Anxiety/ Depression | **Beck’s Anxiety/ Depression Inventories** | 1 & 2 | S | S |

Note: S=Self-report (i.e. participant); P=Parent-report.

Supplementary Table 2.

*Descriptives and group comparisons for participant characteristics and alexithymia at T2.*

| a) Demographic/ Clinical | ASD (T2) | | | | Non-ASD (T2) | | | | Group Comparison | | |
| --- | --- | --- | --- | --- | --- | --- | --- | --- | --- | --- | --- |
|  | ***N*** | **Median (*IQR*)** | **Range** | ***N*** | | **Median (*IQR*)** | | **Range** | ***X^2^* (_df,_ *_N_*)** | ***p*** | ***φ*** |
| Sex: Males (Females) | 51 (25) | - | - | | 33 (26) | - | - | | 1.76 (_1,135_) | 0.18 | 0.11 |
|  |  |  |  | |  |  |  | | ***Z*** | ***p*** | ***r* effect size** |
| Age (years) | 76 | 20.94 (7.56) | 13-31 | | 59 | 19.72 (6.62) | 14-31 | | 0.91 | 0.36 | 0.08 |
| SRS-2 (Self) | 31 | 65.00 (14.50) | 46-91 | | 27 | 47.00 (9.50) | 39-76 | | 4.32 | <0.001*** | 0.58 |
| SRS-2 (Parent) | 19 | 68.00 (18.00) | 43-90 | | 17 | 42.00 (3.00) | 38-45 | | 4.92 | <0.001*** | 0.82 |
| RBS-R | 58 | 7.00 (7.75) | 0-28 | | 17 | 0.00 (2.00) | 0-6 | | 4.64 | <0.001*** | 0.54 |
| Beck’s Anxiety | 74 | -0.04 (1.17) | -1.11-4.39 | | 58 | -0.62 (0.71) | -1.02-1.46 | | 4.16 | <0.001*** | 0.36 |
| Beck’s Depression | 74 | 0.05 (1.53) | -0.92-4.75 | | 58 | -0.51 (0.56) | -0.92-1.92 | | 3.86 | <0.001*** | 0.34 |
| b) Alexithymia | **ASD (T2)** | | | | **Non-ASD (T2)** | | | | **Group Comparison** | | |
|  | ***N*** | **Median (*IQR*)** | **Range** | | ***N*** | **Median (*IQR*)** | **Range** | | ***Z*** | ***p*** | ***r* effect size** |
| TAS-20 Total | 76 | 48.00 (18.25) | 20-93 | | 59 | 38.00 (9.50) | 23-66 | | 4.99 | <0.001*** | 0.43 |
| TAS-20 Identify | 76 | 14.00 (11.25) | 7-35 | | 59 | 9.00 (5.50) | 7-23 | | 4.28 | <0.001*** | 0.37 |
| TAS-20 Describe | 76 | 14.00 (7.00) | 5-24 | | 59 | 10.00 (5.00) | 5-23 | | 4.88 | <0.001*** | 0.42 |
| TAS-20 External | 76 | 21.00 (6.25) | 8-34 | | 59 | 19.00 (7.00) | 10-27 | | 1.77 | 0.08 | 0.15 |

Note: SRS-2=Social Responsiveness Scale – Second Edition; RBS-R=Repetitive Behaviour Scale – Revised; TAS=Toronto Alexithymia Scale; IQR=Interquartile range; *X^2^* (_df,_ *_N_*)=Chi-square test (degrees of freedom, number of participants); *Z*=statistic for Mann-Whitney comparison, *r*=effect size (*Z*/√*N*). ****p*<0.01 (significant after Bonferroni correction; *p*=0.05/11).

Supplementary Table 3.

Bivariate correlations between T1 and T2 measures in the: a) whole sample; b) ASD group.

| 1. All | | T1 | | | | | | | | | T2 | | | | | | | |
| --- | --- | --- | --- | --- | --- | --- | --- | --- | --- | --- | --- | --- | --- | --- | --- | --- | --- | --- |
|  |  | **1** | **2** | **3** | **4** | **5** | **6** | **7** | **8** | **9** | **10** | **11** | **12** | **13** | **14** | **15** | **16** | **17** |
| T1 | **1. TAS Total** | - | - | - | - | - | - | - | - | - | - | - | - | - | - | - | - | - |
|  | **2. TAS Identify** | **0.80***** | - | - | - | - | - | - | - | - | - | - | - | - | - | - | - | - |
|  | **3. TAS Describe** | **0.88***** | **0.66***** | - | - | - | - | - | - | - | - | - | - | - | - | - | - | - |
|  | **4. TAS External** | **0.57***** | 0.14** | **0.33***** | - | - | - | - | - | - | - | - | - | - | - | - | - | - |
|  | **5. SRS-2 (Self)** | **0.79***** | **0.70***** | **0.73***** | **0.35***** | - | - | - | - | - | - | - | - | - | - | - | - | - |
|  | **6. SRS-2 (Parent)** | **0.46***** | **0.37***** | **0.41***** | **0.33***** | **0.59***** | - | - | - | - | - | - | - | - | - | - | - | - |
|  | **7. RBS-R** | **0.45***** | **0.37***** | **0.39***** | **0.26***** | **0.59***** | **0.81***** | - | - | - | - | - | - | - | - | - | - | - |
|  | **8. Beck’s Anxiety** | **0.55***** | **0.65***** | **0.47***** | 0.08 | **0.64***** | **0.36***** | **0.34***** | - | **-** | - | - | - | - | - | - | - | - |
|  | **9. Beck’s Depression** | **0.55***** | **0.64***** | **0.49***** | 0.08 | **0.66***** | **0.38***** | **0.32***** | **0.79***** | **-** | **-** | - | - | - | - | - | - | - |
| T2 | **10. TAS Total** | **0.66***** | **0.47***** | **0.57***** | **0.44***** | **0.63***** | 0.33** | 0.25* | **0.44***** | **0.44***** | - | - | - | - | - | - | - | - |
|  | **11. TAS Identify** | **0.55***** | **0.64***** | **0.47***** | 0.11 | **0.57***** | 0.29** | 0.20 | **0.54***** | **0.51***** | **0.78***** | - | - | - | - | - | - | - |
|  | **12. TAS Describe** | **0.65***** | **0.46***** | **0.63***** | **0.40***** | **0.61***** | 0.31** | 0.27** | **0.41***** | **0.42***** | **0.87***** | **0.66***** | - | - | - | - | - | - |
|  | **13. TAS External** | **0.35***** | 0.02 | 0.26** | **0.56***** | 0.28** | 0.17 | 0.12 | 0.08 | 0.10 | **0.66***** | 0.21* | **0.37***** | - | - | - | - | - |
|  | **14. SRS-2 (Self)** | **0.75***** | **0.59***** | **0.65***** | **0.54***** | **0.81***** | 0.37* | 0.23 | **0.50***** | **0.51***** | **0.69***** | **0.63***** | **0.66***** | 0.35** | - | - | - | - |
|  | **15. SRS-2 (Parent)** | **0.57***** | **0.49***** | **0.48***** | 0.42** | **0.75***** | **0.91***** | **0.78***** | **0.48***** | **0.47***** | 0.48** | 0.52** | 0.35* | 0.16 | 0.03 | - | - | - |
|  | **16. RBS-R** | **0.49***** | **0.48***** | **0.43***** | 0.31** | **0.59***** | **0.76***** | **0.81***** | **0.54***** | **0.43***** | 0.39** | 0.34** | 0.32** | 0.22 | 0.28 | **0.80***** | - | - |
|  | **17. Beck’s Anxiety** | **0.38***** | **0.60***** | 0.26** | 0.01 | **0.47***** | **0.40***** | 0.37** | **0.71***** | **0.61***** | **0.34***** | **0.52***** | 0.29** | -0.04 | 0.38** | 0.47** | 0.34** | - |
|  | **18. Beck’s Depression** | **0.38***** | **0.47***** | **0.32***** | 0.09 | **0.47***** | 0.31** | 0.23* | **0.58***** | **0.64***** | **0.34***** | **0.42***** | 0.30** | 0.09 | 0.34** | 0.40** | 0.32** | **0.71***** |
| 1. ASD | | **T1** | | | | | | | | | **T2** | | | | | | | |
|  |  | **1** | **2** | **3** | **4** | **5** | **6** | **7** | **8** | **9** | **10** | **11** | **12** | **13** | **14** | **15** | **16** | **17** |
| T1 | **1. TAS Total** | - | - | - | - | - | - | - | - | - | - | - | - | - | - | - | - | - |
|  | **2. TAS Identify** | **0.82***** | - | - | - | - | - | - | - | - | - | - | - | - | - | - | - | - |
|  | **3. TAS Describe** | **0.82***** | **0.60***** | - | - | - | - | - | - | - | - | - | - | - | - | - | - | - |
|  | **4. TAS External** | **0.45***** | 0.01 | 0.18** | - | - | - | - | - | - | - | - | - | - | - | - | - | - |
|  | **5. SRS-2 (Self)** | **0.74***** | **0.67***** | **0.68***** | 0.20** | - | - | - | - | - | - | - | - | - | - | - | - | - |
|  | **6. SRS-2 (Parent)** | 0.23** | 0.08 | 0.11 | **0.38***** | 0.22** | - | - | - | - | - | - | - | - | - | - | - | - |
|  | **7. RBS-R** | 0.26** | 0.16 | 0.17* | 0.26** | 0.30** | **0.66***** | - | - | - | - | - | - | - | - | - | - | - |
|  | **8. Beck’s Anxiety** | **0.48***** | **0.61***** | **0.29***** | 0.01 | **0.55***** | 0.16 | 0.16 | **-** | **-** | - | - | - | - | - | - | - | - |
|  | **9. Beck’s Depression** | **0.49***** | **0.59***** | **0.31***** | 0.03 | **0.57***** | 0.12 | 0.06 | **0.72***** | **-** | **-** | - | - | - | - | - | - | - |
| T2 | **10. TAS Total** | **0.63***** | **0.47***** | **0.53***** | 0.37** | **0.46***** | 0.11 | <0.01 | 0.38** | 0.36** | - | - | - | - | - | - | - | - |
|  | **11. TAS Identify** | **0.56***** | **0.66***** | **0.43***** | 0.07 | **0.50***** | 0.10 | -0.06 | **0.50***** | **0.43***** | **0.82***** | - | - | - | - | - | - | - |
|  | **12. TAS Describe** | **0.55***** | 0.34** | **0.57***** | 0.29** | **0.41***** | 0.07 | -0.03 | 0.21 | 0.31** | **0.85***** | **0.61***** | - | - | - | - | - | - |
|  | **13. TAS External** | 0.40** | 0.09 | 0.30** | **0.60***** | 0.22 | 0.20 | 0.18 | 0.16 | 0.12 | **0.64***** | 0.22* | 0.39** | - | - | - | - | - |
|  | **14. SRS-2 (Self)** | **0.83***** | **0.64***** | **0.79***** | 0.38* | **0.78***** | 0.05 | -0.02 | 0.52** | **0.61***** | **0.66***** | **0.68***** | **0.74***** | 0.26 | - | - | - | - |
|  | **15. SRS-2 (Parent)** | 0.57** | 0.51** | 0.38* | 0.43* | **0.61***** | **0.73***** | 0.27 | 0.60** | 0.36* | 0.57** | 0.57** | 0.40 | 0.39 | 0.26 | - | - | - |
|  | **16. RBS-R** | **0.44***** | 0.36** | 0.32** | **0.39***** | **0.40***** | **0.59***** | **0.71***** | **0.47***** | 0.29** | 0.22 | 0.11 | 0.11 | 0.30* | 0.21 | 0.57** | - | - |
|  | **17. Beck’s Anxiety** | 0.39** | **0.61***** | 0.21 | -0.003 | 0.38** | 0.18 | 0.19 | **0.64***** | **0.60***** | 0.21 | **0.43***** | 0.08 | -0.08 | 0.24 | 0.31 | 0.16 | - |
|  | **18. Beck’s Depression** | 0.41** | **0.51***** | 0.29** | 0.11 | 0.41** | 0.19 | 0.08 | **0.52***** | **0.70***** | 0.21 | 0.30** | 0.20 | 0.02 | 0.23 | 0.42 | 0.19 | **0.69***** |

Note: TAS=Toronto Alexithymia Scale; SRS-2=Social Responsiveness Scale – Second Edition; RBS-R=Repetitive Behaviour Scale – Revised. **p*<0.05; ***p*<0.01; ****p*<0.0002 (significant after Bonferroni correction; *p*=0.05/306).

Supplementary Table 4.

Descriptives and group comparison of social-communication difficulties, anxiety and depression symptoms for those with ‘severe’ (≥61) vs. lower levels of alexithymia in the: a) whole sample; b) ASD group.

| a) All | Severe alexithymia (≥61) | | | Lower Alexithymia (<61) | | | Group-comparison | | |
| --- | --- | --- | --- | --- | --- | --- | --- | --- | --- |
|  | ***N*** | **Median (*IQR*)** | **Range** | ***N*** | **Median (*IQR*)** | **Range** | ***Z*** | ***p*** | ***r* effect size** |
| SRS-2 (Self) | 56 | 70.00 (11.00) | 47-94 | 263 | 50.00 (14.50) | 37-85 | 9.82 | <0.001*** | 0.55 |
| SRS-2 (Parent) | 46 | 70.00 (21.00) | 44-95 | 160 | 58.00 (25.00) | 37-90 | 4.73 | <0.001*** | 0.33 |
| Beck’s Anxiety | 53 | 0.86 (1.59) | -0.81-4.65 | 265 | -0.46 (0.97) | -1.28-2.63 | 8.02 | <0.001*** | 0.45 |
| Beck’s Depression | 52 | 1.10 (1.75) | -0.86-4.15 | 265 | -0.43 (0.82) | -1.08-3.42 | 7.56 | <0.001*** | 0.42 |
| b) ASD | **Severe alexithymia (≥61)** | | | **Lower Alexithymia (<61)** | | | **Group-comparison** | | |
|  | ***N*** | **Median (*IQR*)** | **Range** | ***N*** | **Median (*IQR*)** | **Range** | ***Z*** | ***p*** | ***r* effect size** |
| SRS-2 (Self) | 50 | 71.00 (9.00) | 57-94 | 123 | 59.00 (10.50) | 40-85 | 7.99 | <0.001*** | 0.61 |
| SRS-2 (Parent) | 42 | 72.00 (19.25) | 44-95 | 100 | 67.00 (18.00) | 43-90 | 2.56 | 0.01*** | 0.22 |
| Beck’s Anxiety | 46 | 1.04 (1.79) | -0.81-4.65 | 122 | -0.07 (1.09) | -1.28-2.63 | 6.20 | <0.001*** | 0.48 |
| Beck’s Depression | 45 | 1.14 (1.91) | -0.75-4.15 | 123 | -0.21 (0.94) | -0.97-3.42 | 5.97 | <0.001*** | 0.46 |

Note: SRS-2=Social Responsiveness Scale – Second Edition; IQR=Interquartile range; *Z*=statistic for Mann-Whitney comparison; *r*=effect size (*Z*/√*N*). ****p*<0.01 (significant after Bonferroni correction; *p*=0.05/8).
